# Getting a Grip on the Handgrip Task: Handgrip Duration Correlates with Neuroticism But Not Conscientiousness

**DOI:** 10.3389/fpsyg.2017.01367

**Published:** 2017-08-11

**Authors:** Simon B. Goldberg, Lisa Flook, Matthew J. Hirshberg, David Findley, Pelin Kesebir, Stacey M. Schaefer, Richard J. Davidson

**Affiliations:** ^1^Center for Healthy Minds, University of Wisconsin–Madison, Madison WI, United States; ^2^Department of Counseling Psychology, University of Wisconsin–Madison, Madison WI, United States; ^3^Department of Educational Psychology, University of Wisconsin–Madison, Madison WI, United States; ^4^Waisman Laboratory for Brain Imaging and Behavior, University of Wisconsin–Madison, Madison WI, United States; ^5^Department of Psychology, University of Wisconsin–Madison, Madison WI, United States

**Keywords:** handgrip task, self-regulation, self-control, neuroticism, conscientiousness

## Abstract

Questions regarding the replicability of key findings in the self-regulation literature (e.g., ego-depletion effect) have led some to call for a more thorough evaluation of commonly used measures of self-control. The isometric handgrip task is one such measure. The current study examined correlates of handgrip persistence using data drawn from a larger randomized controlled trial. Handgrip persistence was measured both at baseline and following a physical stressor (cold pressor test). Correlations were examined between handgrip performance and personality traits theoretically closely linked with self-regulation: conscientiousness and neuroticism. Baseline handgrip performance was correlated with several measures drawn from the nomological network of self-regulation including measures of trait neuroticism, mindfulness, anxiety sensitivity, perceived stress, and positive affect, although not with trait conscientiousness. Baseline handgrip predicted aversiveness experienced during the physical stressor, while changes in handgrip performance tracked changes in implicit and explicit negative affect (i.e., affective reactivity). These associations were largely maintained when controlling for variables highly correlated with overall grip strength (i.e., gender, height, and weight), although correlations separated by gender suggest associations were primarily driven by female participants. Results support future research using the handgrip task.

## Introduction

The ability to regulate one’s behavior is vital for daily life. Self-regulation has been defined as “the exertion of control over the self by the self” (Muraven and Baumeister, 2000) and a substantial body of research has documented the importance of self-regulation for a wide range of life outcomes (e.g., grade point average, binge eating, alcohol abuse, management of chronic pain; Tangey et al., 2004; Nes et al., 2009). The ego-depletion paradigm is one method that has been used experimentally to study self-control. This paradigm typically involves assessing the negative impact of engaging in tasks that theoretically consume self-control resources on performance of subsequent self-control tasks (i.e., the sequential task paradigm; Baumeister et al., 1998). These studies are based on the theoretical notion that self-control relies upon limited physiological and cognitive resources (i.e., limited strength model of self-control; Baumeister et al., 1998). The ego-depletion effect has been established via meta-analysis (*k* = 83 studies, *d* = 0.62), with evidence that numerous factors can promote or inhibit one’s ability to engage in self-regulatory behaviors (e.g., perceived difficulty, subjective fatigue, effort, blood glucose levels; Hagger et al., 2010).

However, concerns have recently been raised about this body of literature. More recent evidence suggests that Hagger et al. (2010) meta-analysis may have over-estimated the experimental effects of ego-depletion, owing primarily to publication bias and the influence of small sample studies (Carter et al., 2015). A recent large, multisite replication study (*N* = 2,141) failed to detect an effect of ego-depletion (Hagger et al., 2016).

Amidst concerns regarding the replicability of the ego-depletion effect, some have voiced misgivings regarding the theoretical and methodological basis of self-control research generally. Lurquin and Miyake (2017) express several areas of “conceptual crisis,” including what they view as an inadequate operationalization of self-control and a lack of empirical validation of self-control tasks. Lurquin and Miyake note the potential of correlational research as a method for establishing what precisely is measured by tasks purported to index self-control.

The isometric handgrip task is a commonly used measure of self-regulation. This task involves clenching the hand muscles around an object, typically a hand dynamometer (capable of measuring force) or a spring-loaded exercise handgrip. The device is often calibrated based on an individual’s overall handgrip strength (e.g., at 50% of maximum grip strength; Stork et al., 2016), which makes the task difficulty uniform across subjects with varying grip strength. When used as a pre–post measure of self-regulation, a single, non-calibrated device is often used for all participants (e.g., Muraven et al., 1998). The duration that an individual keeps the device closed is theorized to represent a relatively pure measure of self-regulation, with one’s ability to continue squeezing the grip primarily dependent on one’s ability to overcome the associated fatigue and “pushing oneself to continue” (Ciarocco et al., 2001, p. 1160). Experimental effects on handgrip persistence have been demonstrated using ego-depletion paradigms. Hagger et al. (2010) report a moderate sized effect on this measure (*d* = 0.64, *k =* 18 effect sizes), with Carter et al. (2015) reporting a trim-and-fill adjusted effect of *g* = 0.36 (although, of note, this effect was non-significant when adjusted for small-study effects).

Despite widespread use of the handgrip task in experimental research, to our knowledge no studies have explored the broader psychological correlates of handgrip performance. Given questions regarding validity of self-control tasks generally (Lurquin and Miyake, 2017), it is worthwhile examining the degree to which handgrip performance correlates with psychological characteristics theoretically linked to self-regulation. While one recent study reports that performance on this task predicted exercise and academic behavior (Stork et al., 2016), the relationship between handgrip task performance and personality variables is unknown.

Of the Big Five personality dimensions, self-regulation has been most closely tied to conscientiousness and neuroticism (Tangey et al., 2004; McCrae and Löckenhoff, 2010; Friedman and Kern, 2014). Impulsivity, for example, is a classic failure of self-regulation that is linked to high neuroticism and low conscientiousness (McCrae and Löckenhoff, 2010). Substance use is associated with both self-regulatory deficits (Tangey et al., 2004) as well as high neuroticism and low conscientiousness (Kotov et al., 2010).

In order to examine the broader nomological net in which self-regulation is situated (as is recommended when examining the construct validity of a given measure; Cronbach and Meehl, 1955), we aimed to assess several constructs that have been previously linked with neuroticism and conscientiousness (i.e., tests of convergent validity). Neuroticism has been associated with susceptibility to stress and measures of affect (Friedman and Kern, 2014), as well as with affective reactivity (Gross et al., 1998). Both neuroticism and conscientiousness have been linked in the expected direction to measures of mindfulness (which involves self-regulation of attention; Thompson and Waltz, 2007; Giluk, 2009). Conscientiousness has been inversely linked with depressive disorders (Kotov et al., 2010) which are themselves characterized by high negative affect and a lack of positive affect.

In addition, no research to our knowledge has explored the potential impact of gender on the link between handgrip duration and personality variables (i.e., gender as a moderator). Potential gender differences in handgrip performance have rarely been discussed, although there is evidence that gender may predict handgrip duration (Howells, 1933; West et al., 1995) and gender has been associated with neuroticism (albeit weakly; Hyde, 2014). It is possible that certain self-regulation tasks function differently for males and females (especially tasks with physical strength demands), although the direction of this difference is unknown.

The current study explores the broader personality correlates of the handgrip task as a preliminary attempt to broaden its construct validity (i.e., establish what is being measured; Lurquin and Miyake, 2017). To do so, we examined the correlation of handgrip task performance with measures drawn from the nomological network of self-regulation, namely trait neuroticism, trait conscientiousness, trait mindfulness, susceptibility to stress, and affect. To do so, we used data drawn from a larger randomized controlled trial (RCT). This trial examined the impact of three brief contemplative practices (mindfulness, loving kindness, and gratitude) to buffer against the effects of the cold pressor test (CPT), a standardized physical stressor (Lovallo, 1975). Inclusion of this stressor allowed examination between baseline handgrip performance and ratings of the aversiveness of the stressor. In addition, changes in handgrip performance and changes in affect pre- and post-stressor were examined, in keeping with evidence that neuroticism is linked with affective reactivity (Gross et al., 1998).

The following hypotheses guided this work:

*H1:* Handgrip performance will be positively correlated with trait conscientiousness and measures drawn from the nomological network of trait conscientiousness (e.g., mindfulness).
*H2*: Handgrip performance will be negatively correlated with trait neuroticism and measures drawn from the nomological network of trait conscientiousness (e.g., stress susceptibility).

As data were drawn from a larger RCT, it was important to examine whether handgrip performance was impacted by group assignment. We had no *a priori* hypotheses regarding differential treatment effects on handgrip performance. In addition, we examined whether the relationship between handgrip performance and personality was moderated by gender. Based on a lack of literature related to gender as a moderator of task performance, we had no *a priori* directional hypothesis.

## Materials and Methods

### Participants

Data for the current study were drawn from a RCT comparing the effects of three brief mental trainings and an attentional control on measures of self-regulation and executive function following a physical challenge (CPT). The CPT was used in order to elicit a physiological stress response (Lovallo, 1975). One hundred and fifty-six undergraduate students (97 females and 59 males) participated in this study in exchange for extra credit for their psychology course. Participants were informed they would be participating in a study examining the effects of brief trainings on cognition and behavior and that the experiment would involve submerging their hands in an ice water bath for 3 min. Sample racial demographics were representative of the university population: White (83.3%), Asian (5.8%), Black or African American (3.8%), more than one race (5.8%), unknown or did not report (1.3%). Sample ethnic identities were: Hispanic or Latino (7.7%), not Hispanic or Latino (86.5%), unknown or did not report (5.8%). Participants were on average 19.29 years old (*SD* = 0.74) and had completed 2.29 semesters of college (*SD* = 1.30).

### Interventions

#### Brief Trainings

Following baseline assessment, participants were randomly assigned to listen to one of four 12- to 15-min recordings. Three of these were based on contemplative practices (mindfulness, loving-kindness, gratitude; Wood et al., 2010; Hofmann et al., 2011; Khoury et al., 2013) and one was designed as an attentional control condition (describing and then picturing the inside of a familiar building in detail). Participants were provided headphones and were asked to follow the instructions given in the recording. Further details of this trial and the four training conditions have been reported elsewhere (Schaefer et al., unpublished).

### Materials

#### Baseline Self-report Measures

Several standardized self-report questionnaires were collected at baseline, prior to both the training and CPT in order to examine correlates of handgrip duration performance with measures related to conscientiousness and neuroticism. Measures of personality, attention regulation, susceptibility to stress, and affect were included, along with a demographic questionnaire (**Table 1**).

**Table 1:** Baseline and post-stressor variables descriptive statistics.

	Full sample		Females		Males	
	Mean	*SD*	Mean	*SD*	Mean	*SD*
% Female	0.62	0.49	1	0	0	0
Age (years)	19.29	0.74	19.26	0.79	19.35	0.67
Semesters of college	2.29	1.3	2.27	1.35	2.32	1.22
Height (inches)	67.86	4.23	65.37	2.77	71.94	2.79
Weight (lbs)	153.96	31.04	139.42	21.86	177.85	29.15
BFI neuroticism	3.2	0.82	3.41	0.79	2.86	0.74
BFI conscientiousness	4.27	0.61	4.31	0.61	4.22	0.62
MAAS	3.9	0.82	3.85	0.82	3.99	0.8
Anxiety sensitivity	2.25	0.64	2.38	0.68	2.05	0.5
Perceived stress	2.79	0.46	2.87	0.48	2.66	0.41
PANAS positive	2.75	0.69	2.67	0.67	2.87	0.71
PANAS negative	1.32	0.32	1.33	0.32	1.29	0.31
IPANAT positive	2.08	0.42	2.11	0.41	2.04	0.43
IPANAT negative	1.87	0.4	1.84	0.39	1.9	0.4
Handgrip duration (seconds)	40.87	41.05	26.79	26.97	64.02	49.22
CPT aversiveness	71.06	23.3	73.21	21.34	67.52	26.02

*SD, standard deviation; BFI, Big Five Inventory; Mindfulness, Mindful Attention Awareness Scale; Anxiety Sensitivity, Anxiety Sensitivity Scale; Perceived Stress, Perceived Stress Scale; PANAS, Positive and Negative Affect Schedule; Pos, Positive, Neg, Negative; IPANAT, Implicit Positive and Negative Affect Test; CPT, Cold pressor test. *n =* 156 (97 female and 59 male) for all variables except CPT aversiveness where *n* = 140 (87 female, 53 male, includes CPT completers only).*

##### Personality

The nine-item conscientiousness and eight-item neuroticism subscales were used (αs = 0.78, 0.82, for conscientiousness and neuroticism, respectively) from the Big Five Inventory (BFI; John et al., 2008). Sample items include “I am someone who does a thorough job” (conscientiousness) and “I am someone who is emotionally stable, not easily upset” (neuroticism). Higher scores indicate a higher level of the given personality trait.

##### Attention regulation

The Mindful Attention Awareness Scale (MAAS; Brown and Ryan, 2003) is a 15-item measure designed to assess participants’ tendency to engage in mindful attention during their daily life (e.g., “I rush through activities without being really attentive to them,” item is reverse scored). Internal consistency was acceptable in the current sample (α = 0.88).

##### Susceptibility to stress

Two measures assessed participants’ baseline susceptibility to stress. The Anxiety Sensitivity Index (ASI; Reiss et al., 1986) is a 16-item measure used to assess individuals’ “fear of fear” (Reiss et al., 1986, p.1; e.g., “it is important to me not to appear nervous”). The measure showed acceptable internal consistency in the current sample (α = 0.87). The Perceived Stress Scale (PSS; Cohen et al., 1983) is a 10-item measure that was used to assess participants’ appraisal of their life situations as stressful in the past month (e.g., “how often have you felt that you were unable to control the important things in your life?”). Internal consistency was adequate in the current sample (α = 0.78).

##### Demographic and physical characteristics

Participants completed a brief questionnaire reporting their age, gender, number of semesters of college completed, race/ethnicity, height, and weight.

#### Post-stressor Ratings

A three-item scale based on prior research (Raio et al., 2013) was used to assess subjective aversiveness of the CPT as a measure of affective reactivity (which has been linked with neuroticism; Gross et al., 1998). Participants used a rating scale (0 to 100) to indicate how “stressful,” “painful,” and “unpleasant” they found the CPT. The mean of these three items was used as an overall indicator of aversiveness of the stressor (α = 0.82).

#### Pre–Post Self-report Measures

Measures of both explicit and implicit current positive and negative affect were used at baseline and following the CPT, also indexing affective reactivity.

##### PANAS

The 20-item Positive and Negative Affect Schedule (PANAS; Watson et al., 1988) was used to assess participants’ current positive (e.g., “interested”) and negative (e.g., “distressed”) affect at baseline and following the CPT test. Internal consistency was adequate in the current sample for both positive (α = 0.87) and negative (α = 0.71) subscales.

##### Implicit positive and negative affect test (IPANAT; Quirin et al., 2009)

A 36-item measure of implicit positive and negative affect was used. This measure asks participants to rate the extent to which non-sense words (e.g., “tunba”) convey various positively or negatively valenced adjectives (e.g., “cheerful,” “helpless”). The measure has shown adequate test–retest reliability and appears to reflect state variance in affect (Quirin et al., 2009). Internal consistency was adequate in the current sample for both positive (α = 0.75) and negative (α = 0.74) items.

#### Handgrip Task

The handgrip task was used at baseline and following the CPT test. The procedure was designed following previous studies employing this task (e.g., Muraven et al., 1998). Participants were given a commercially available exercise handgrip (HHG-GG001, Gold’s Gym, Dallas, TX, United States), told to hold it in their dominant hand, and were asked to squeeze the handgrip a few times first to assess its tension. The apparatus requires 9.07 kg (88.96 N) of force to close, which is at or below approximately 30% of the average maximal grip strength for males and females within our sample’s age range (Massy-Westropp et al., 2004; Günther et al., 2008). A single device was used for simplicity of administration, but has the drawback of creating a task that varied in difficulty across participants (in contrast to using a handgrip device that is calibrated based on individuals’ overall grip strength; Stork et al., 2016). An approximately one-inch thick block of wood was placed in the center of the handgrip and participants were told to hold the block of wood with the handgrip for as long as possible. Using a block allowed researchers to more accurately determine when participants’ grip loosened (i.e., when the block fell). The block also further decreased the physical demands of the task. The combination of the modest amount of force required to close the grip in combination with the block made the task less reliant on participants’ overall grip strength (Mehta and Cavuoto, 2015). Handgrip holding times were log-transformed to account for a positive skew in the distribution. As the current study did not measure overall grip strength nor calibrate the handgrip based on grip strength, models are reported controlling for variables shown to be highly correlated with grip strength (i.e., gender, height, weight; Chatterjee and Chowdhuri, 1991; Budziareck et al., 2008; Günther et al., 2008).

#### Cold Pressor Test (CPT; Lovallo, 1975)

The CPT is a standardized physical stressor that has been widely used and shown to be of relatively low risk to a range of participants, including young children (von Baeyer et al., 2005). The task has been shown to elicit autonomic stress reactivity (including increased cortisol production) as well as affective reactivity (Raio et al., 2013). In the current study, participants were asked to keep their non-dominant hand in an ice bath for 3 min. Participants were dropped from post-test analyses (i.e., correlations with ratings of aversiveness, with pre–post changes in affect) if they did not complete the full 3 min. Sixteen participants were dropped for this reason.

### Procedure

Upon presenting at the laboratory, participants were randomized to one of the four conditions (mindfulness, loving kindness, gratitude, or attentional control). Participants and experimenters were blind to group assignment. Participants then completed a battery of self-report questionnaires and the handgrip task. Next, participants listened to their respective training. Following the training, participants completed the CPT, the CPT aversiveness ratings, post-test ratings of explicit and implicit affect (i.e., PANAS and IPANAT), and the handgrip task once again.

### Statistical Analysis

Analysis of variance (ANOVA) models were used to assess differences in baseline handgrip performance (one-way ANOVA) and differential changes in handgrip performance by group assignment (time by group interaction, i.e., two-way ANOVA). The primary study hypotheses were tested with correlations between handgrip performance and measures related to conscientiousness and neuroticism. Subsequent regression models controlled for variables potentially correlated with grip strength (e.g., gender and weight). Regression models were also constructed assessing potential moderation of the link between handgrip performance and personality by gender. These models added the interaction between gender and baseline handgrip as predictors of personality variables.

## Results and Discussion

Intervention groups did not differ on baseline handgrip duration [*F*(3,152) *=* 1.29, *p* = 0.281] nor was a significant time by group interaction observed in changes in handgrip [*F*(3,303) *=* 0.20, *p* = 0.896]. As the focus of the current study was on examining correlates of the handgrip task and groups did not differ on handgrip performance, group assignment was ignored in all further analyses.

Correlations were computed between baseline handgrip persistence, baseline psychological variables, and CPT aversiveness ratings. As shown in **Table 2**, longer baseline handgrip duration was associated with lower neuroticism as well as with numerous measures linked to lower neuroticism and higher conscientiousness in previous studies including higher mindfulness, lower anxiety sensitivity, lower perceived stress, higher explicit positive affect, and lower CPT aversiveness. Contrary to expectations, baseline handgrip was not correlated with trait conscientiousness (*r* = 0.04, *p* = 0.615).

**Table 2:** Correlations between handgrip performance and individual difference variables.

Variable	*r*	*r^a^*	*r* females	*r* males
BFI neuroticism	–0.38^∗∗∗^	–0.25^∗∗^	–0.34^∗∗^	–0.09
BFI conscientiousness	0.04	0.08	0.10	0.07
MAAS	0.19^∗^	0.18^∗^	.27^∗∗^	–0.04
Anxiety sensitivity	–0.29^∗∗∗^	–0.20^∗^	–0.29^∗∗^	0.08
Perceived stress	–0.28^∗∗∗^	–0.19^∗^	–0.29^∗∗^	0.01
PANAS positive	0.19^∗^	0.15*^t^*	0.16	0.11
PANAS negative	–0.14*^t^*	–0.12	–0.20^∗^	0.03
IPANAT positive	–0.03	–0.02	–0.05	0.14
IPANAT negative	0.04	0.03	–0.09	0.21
CPT aversiveness	–0.23^∗∗^	–0.20^∗^	–0.24^∗^	–0.15
Δ PANAS positive	0.13	0.17^∗^	0.17	0.15
Δ PANAS negative	–0.18^∗^	–0.17^∗^	–0.09	–0.27^∗^
Δ IPANAT positive	0.07	0.10	0.06	0.14
Δ IPANAT negative	–0.33^∗∗∗^	–0.32^∗∗∗^	–0.26^∗^	–0.42^∗∗^

**^a^*Correlations controlling for gender, height, and weight.*

*^*t*^p < 0.10, ^∗^*p* < 0.05, ^∗∗^*p* < 0.01, ^∗∗∗^*p* < 0.001.*

*Δ = Changes in affect. Coefficients represent the correlation between changes in affect and changes in handgrip duration pre- and post-stressor.*

As the current design used a single apparatus for all participants that was not calibrated for participants’ grip strength (and individuals with greater grip strength may show longer persistence due to the task being less taxing), subsequent models controlled for potential confounding variables. Gender, height, and weight have all been shown to significantly predict grip strength with large effects (e.g., *r*s = 0.88 and 0.86 for height and weight, respectively; Chatterjee and Chowdhuri, 1991). In the current sample, gender significantly predicted handgrip duration with males showing significantly longer durations relative to females [*t*(154) = -6.99, *p* < 0.001], as did height (*r* = 0.45, *p* < 0.001) and weight (*r* = 0.39, *p* < 0.001). Baseline handgrip remained significantly correlated in the expected direction with trait neuroticism, mindfulness, anxiety sensitivity, perceived stress, and CPT aversiveness after controlling for gender, height, and weight (**Table 2**).

**FIGURE 1:**
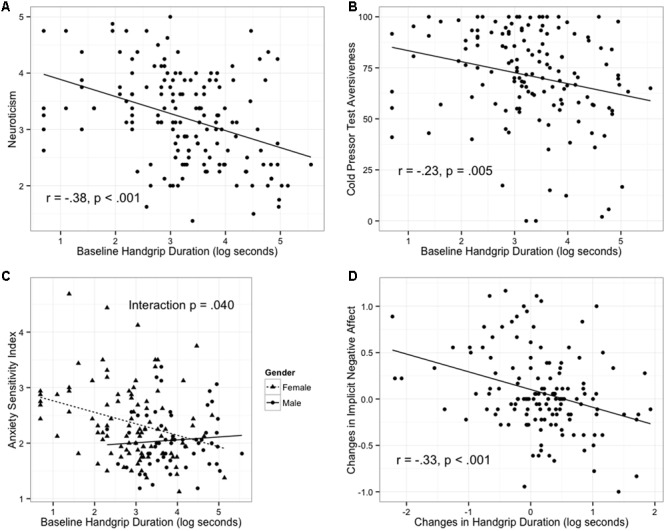
Baseline and change in handgrip duration predict individual differences. **(A)** Baseline handgrip predicts trait neuroticism. **(B)** Baseline handgrip predicts aversiveness in response to cold pressor test (CPT). **(C)** Baseline handgrip predicts anxiety sensitivity for females but not males. **(D)** Changes in handgrip duration (computed as post-test minus pre-test) tracks changes in implicit negative.

Next, correlations were examined between changes in handgrip and changes in explicit and implicit affect. Of note, unlike baseline correlations in which handgrip duration may be confounded with overall grip strength, correlations between change scores were not susceptible to this confound. Increase in handgrip duration from pre- to post-stressor was correlated with reductions in explicit and implicit negative affect (**Table 2** and **Figure 1**) but not with changes in positive affect. These relationships remained unchanged when controlling for gender, height, and weight.

Lastly, regression models were constructed that included an interaction between gender and handgrip duration (baseline or change). These models tested the possibility that the handgrip task may more closely align with psychological variables for either females or males. A significant gender by handgrip interaction was noted for the model predicting anxiety sensitivity (*b* = -0.26, *t* = -2.07, *p =* 0.040) indicating that the relationship between longer baseline handgrip performance and lower anxiety sensitivity is stronger for females than for males (**Figure 1**). No other interaction terms reached significance (*p* > 0.050). Examination of correlations run for female and male participants separately suggests that relationships are primarily driven by female participants (**Table 2**).

The current study examined the correlates of a commonly used behavioral measure of self-regulation, the handgrip duration task, with constructs related to conscientiousness and neuroticism—two personality traits most closely tied to self-regulation (McCrae and Löckenhoff, 2010). This was aimed at addressing the need for more thorough validation of putative measures of self-control (Lurquin and Miyake, 2017) in the context of a replication crisis within the ego-depletion literature (Hagger et al., 2016). Consistent with predictions, baseline performance on this task was shown to correlate with numerous measures closely related to neuroticism. Further, changes in handgrip performance tracked changes in explicit and implicit negative affect from pre- to post-stressor in the expected direction. Contrary to our predictions, however, handgrip performance did not correlate with trait conscientiousness. The observed relationships between handgrip performance and various measures within the nomological network of self-regulation were largely maintained when controlling for variables shown to be associated with grip strength (i.e., gender, height, and weight), while examination of correlations separated by gender suggests females are driving the relationship between baseline handgrip and baseline individual differences although gender only significantly moderated the association in one case (for baseline anxiety). Taken together, these findings suggest that the handgrip task may indeed tap meaningful aspects of personality and individual difference variables related to self-regulation, perhaps most accurately for females.

Examination of the pattern of associations may provide some insight into just what aspects of personality this task is most strongly detecting. While we theorized that both conscientiousness and neuroticism would be linked to handgrip, the pattern of findings suggests that the task may be a stronger predictor of individual variation in neuroticism than conscientiousness. The most robust relationships were noted between handgrip performance and measures associated with negative affectivity, notably trait neuroticism, anxiety sensitivity, perceived stress, and affective reactivity (aversiveness during the CPT, changes in negative affect).

It was contrary to expectations that handgrip performance was not correlated with self-reported trait conscientiousness assessed via the BFI (*r* = 0.04, *p* = 0.615). Examination of correlates between baseline handgrip performance and the individual items composing this subscale of the BFI likewise did not reveal significant associations between particular items and the handgrip (all *p* > 0.050). These findings suggest that the handgrip task may indeed assess individual difference variables relevant to self-regulation, but may index the affective dimension of self-regulation (i.e., emotion regulation and neuroticism) rather than the regulation of behavior in pursuit of goals (i.e., conscientiousness; McCrae and Löckenhoff, 2010).

Strengths of the current study include a reasonably large sample, inclusion of a variety of self-report measures assessing individual differences drawn from the nomological network of self-regulation (including an implicit measure of affect), and inclusion of a standardized stressor. However, several limitations were also present. Perhaps the most striking is the absence of a more extensive measure of self-regulation or self-control. While a recent study suggested that handgrip duration correlates with self-reported self-regulation (Stork et al., 2016), it nonetheless would have been ideal to include a measure such as the Self-control Scale (Tangey et al., 2004) in the current study as well.

A second significant shortcoming in the present study was not calibrating the handgrip resistance based on participants’ overall strength. Our intention was to explore the correlates of a commonly used measure of self-regulation, and thus we employed the task in a manner similar to that used in previous psychological research (e.g., Muraven et al., 1998). Nonetheless, it is conceivable that differences in overall strength may account for some of the observed relationships. Previous research has shown that overall strength is correlated with other health indicators (Rantanen et al., 1994) as well as other aspects of personality (e.g., sensation seeking; Fink et al., 2010). Gender differences in overall strength may also have led to the observed associations. Males and females are known to differ on psychological variables such as neuroticism, sadness, and anxiety, and, while these differences are not large in magnitude (Hyde, 2014), they may explain some of the observed pattern of findings. That said, results were largely maintained in subsequent models that controlled for gender, height, and weight (and height and weight have been shown to correlate strongly with overall grip strength, e.g., *r*s = 0.88 and 0.86; Chatterjee and Chowdhuri, 1991). The fact that several relationships remained when controlling for gender, height, and weight suggests that differences in overall strength are not exclusively driving the results (even though other anthropometric variables known to influence grip strength were not assessed, e.g., hand width and forearm length; Günther et al., 2008). It is also worth noting that changes in handgrip performance (which are not susceptible to the confound of overall grip strength) tracked changes in both explicit and implicit negative affect, supporting the notion that handgrip performance is closely linked with negative affect.

Future research should continue to interrogate the construct validity of the handgrip task. The handgrip task could be included within a battery of measures purported to assess self-regulation (e.g., persistence on unsolvable anagrams, consuming unpleasant-tasting substances; Hagger et al., 2010) to determine the degree of underlying commonality across tasks (as suggested by Lurquin and Miyake, 2017). Work could also productively examine the extent to which handgrip duration is driven by overall grip strength to assess whether or not a single device provides an acceptable alternative to a device calibrated based on an individual’s overall grip strength. One such study would examine relationships between handgrip duration and measures related to self-regulation with and without calibrating the handgrip device as well as with and without controlling for overall grip strength. Commercially available dynamometers that can measure a submaximal static group force (e.g., 30% of an individual’s maximal force) could be used in future studies (e.g., Zona device, Zona Health Inc., Boise, ID, United States).

Demonstrating a correlation between this behavioral measure of self-regulation and self-report measures drawn from the nomological network of self-regulation supports the construct validity of the handgrip task as tapping at least the affective regulation aspect of self-regulation. Findings may also support the notion that targeting negative affect could be a mechanism for enhancing behavioral self-regulation, although future research would need to examine this possibility directly. Given the simplicity of administration, this task may serve as a potentially useful non-self-report method for assessing the affective regulation aspects of self-regulation (particularly if the task is calibrated based on overall grip strength).

## Ethics Statement

This study was carried out in accordance with the recommendations of the American Psychological Association, with written informed consent from all subjects. All subjects gave written informed consent in accordance with the Declaration of Helsinki. The protocol was approved by the University of Wisconsin–Madison Institutional Review Board.

## Author Contributions

SG assisted in study design, analyzed the data, and wrote the first draft of the manuscript. LF, MH, SS, and RD assisted in study design, data analysis and manuscript conceptualization, and edited the manuscript. DF conducted the experiment, assisted in study design, data analysis and manuscript conceptualization, and edited the manuscript. PK assisted in data analysis and manuscript conceptualization, and edited the manuscript.

## Conflict of Interest Statement

RD serves on the board of directors for the following nonprofit organizations: The Mind and Life Institute and the Center for Investigating Healthy Minds, Inc. No donors, either anonymous or identified, have participated in the design, conduct, or reporting of research results in this article. The other authors declare that the research was conducted in the absence of any commercial or financial relationships that could be construed as a potential conflict of interest.
